# A case of pleuroperitoneal communication in which establishing a laparoscopic pneumoperitoneum was useful for the detection of a fistula

**DOI:** 10.1186/s40792-021-01147-1

**Published:** 2021-03-05

**Authors:** Takehiko Manabe, Kenji Ono, Soichi Oka, Yuichiro Kawamura, Toshihiro Osaki

**Affiliations:** Thoracic Surgery, Kokura Memorial Hospital, Asano, Kokurakita-ku, Kitakyushu, Fukuoka 802-8555 Japan

**Keywords:** Pleuroperitoneal communication, Thoracoscopic surgery, Laparoscopic surgery, Pneumoperitoneum, Continuous ambulatory peritoneal dialysis hydrothorax

## Abstract

**Background:**

Pleuroperitoneal communication (PPC) is rarely observed, accounting for 1.6% of all patients who undergo continuous ambulatory peritoneal dialysis (CAPD). Although there have been several reports concerning the management of this condition, we have encountered several cases in which control failed. We herein report a valuable case of PPC in which laparoscopic pneumoperitoneum with video-assisted thoracic surgery (VATS) was useful for supporting the diagnosis and treatment.

**Case presentation:**

The patient was a 58-year-old woman with chronic renal failure due to chronic renal inflammation who was referred to a nephrologist in our hospital to undergo an operation for the induction of CAPD. Post-operatively, she had respiratory failure, and chest X-ray and computed tomography (CT) showed right-sided hydrothorax that decreased when the injection of peritoneal dialysate was interrupted. Therefore, PPC was suspected, and she was referred to our department for surgical repair. We planned surgical treatment via video-assisted thoracic surgery. During the surgery, we failed to detect any lesions with thoracoscopy alone; we therefore added a laparoscopic port at her right-sided abdomen near the navel and infused CO_2_ gas into the abdominal cavity. On thoracoscopy, bubbles were observed emanating from a small pore at the central tendon of the diaphragm, which was considered to be the lesion responsible for the PPC. We closed it by suturing directly.

**Conclusions:**

VATS with laparoscopic pneumoperitoneum should be considered as an effective method for inspecting tiny pores of the diaphragm, especially when the lesions responsible for PPC are difficult to detect.

## Background

The occurrence of hydrothorax due to pleuroperitoneal communication (PPC) under continuous ambulatory peritoneal dialysis (CAPD) is relatively rare, with an incidence of about 1.6% [1]. Although VATS is the standard procedure for the diagnosis and treatment, there are some cases in which disease control failed due to an inability to confirm the lesion responsible during surgery [2]. To our knowledge, there has been no report of surgery using a laparoscopic approach to manage CAPD-related PPC.

We herein report a case in which pneumoperitoneum with laparoscopy during surgery was useful for detecting pinhole PPC.

## Case presentation

**Fig. 1:**
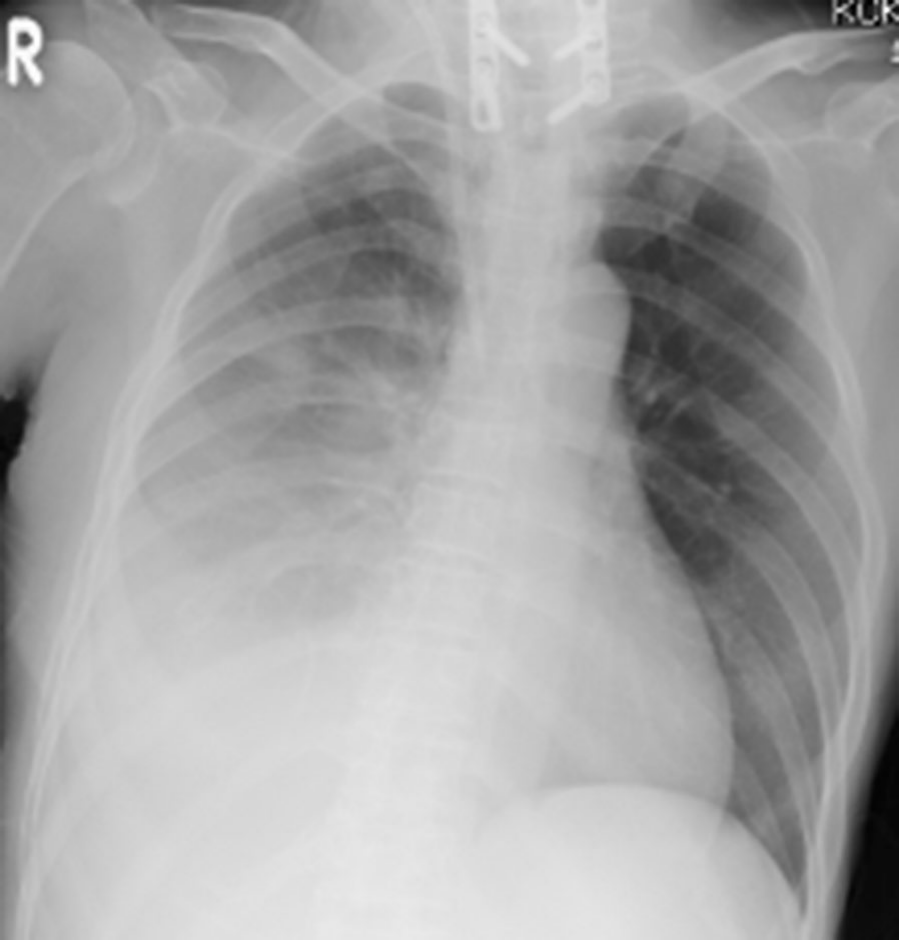
Chest radiograph taken before surgery shows right hydrothorax

The patient was a 58-year-old woman who was referred to a nephrologist in our hospital to undergo an operation for the introduction of CAPD. One week after starting CAPD, she developed hypoxemia and gained weight. Chest X-ray (Fig. 1) and computed tomography showed right hydrothorax. PPC was suspected, as cessation of CAPD improved the hydrothorax.

We planned to perform surgical repair of the diaphragm using video-assisted thoracic surgery (VATS). Dialysis solution (1000 ml) containing 20 mg of indigo carmine was injected into the abdominal cavity through the CAPD catheter just before starting the surgery. Under general anesthesia, the patient was intubated with a double-lumen endotracheal tube and positioned in a left-sided half-lateral decubitus position. Three 1.5-cm skin incisions were made at the seventh, sixth, and eighth intercostal spaces on the anterior, middle, posterior axillary lines, respectively. A 30°, 10-mm thoracoscope was inserted at the sixth intercostal space of the middle axillary line.

**Fig. 2:**
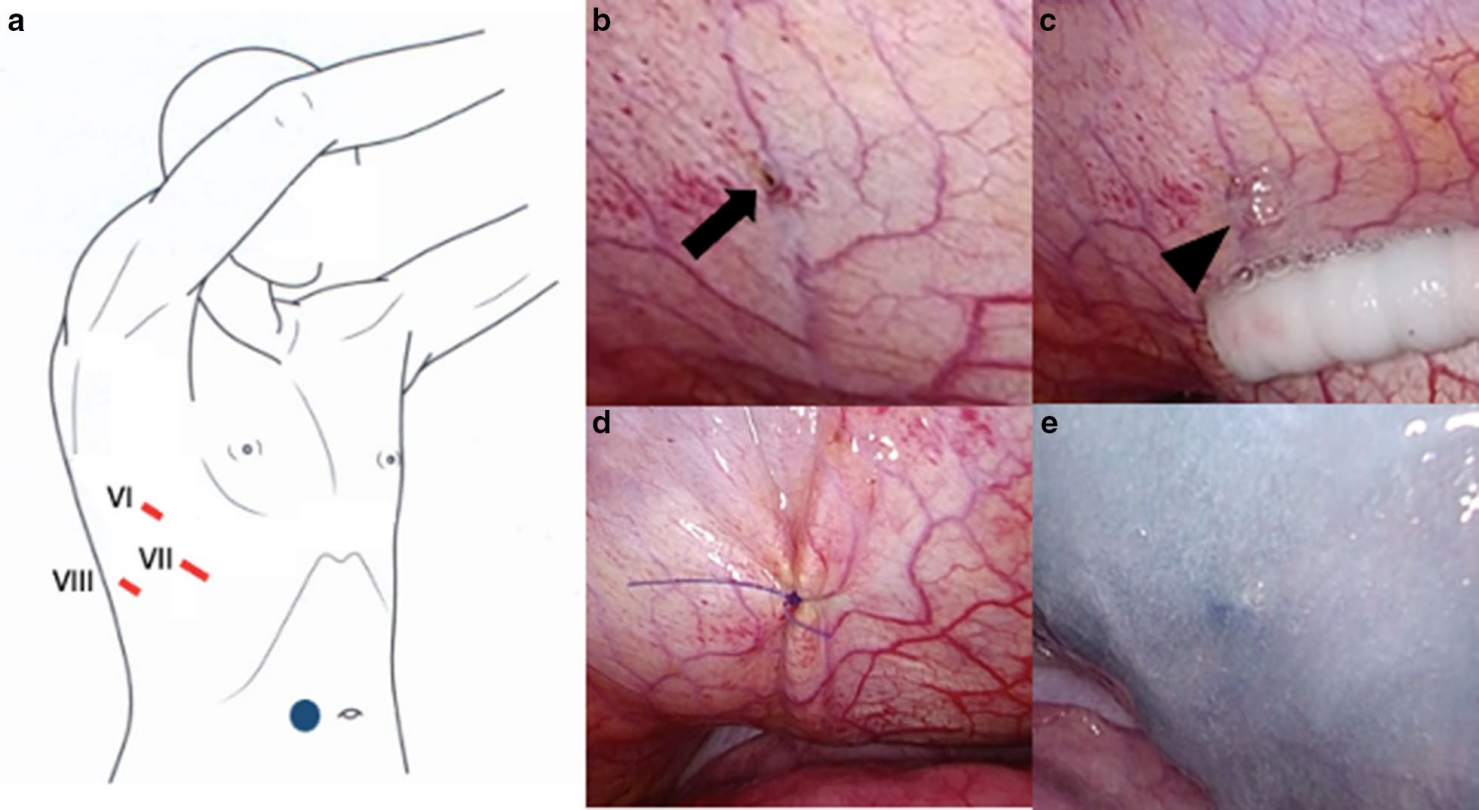
Diagram of the surgery and surgical view. **a** VATS in the left-sided half-lateral decubitus position; one skin incision was made to the right side of the navel, and a laparoscope was inserted into this port (blue round). **b** A thinned diaphragm was detected at the central tendon of the diaphragm (arrow). **c** Bubbles were observed at this small pore after pneumoperitoneum (arrowhead). **d** The small pore was closed with 4-0 non-absorbable monofilament sutures. **e** The central tendon around the reinforcement was covered with a sheet of absorbable polyglycolic acid felt and sprayed with fibrin glue

However, despite the injection of indigo carmine into the abdominal cavity and the careful inspection of the diaphragm with the thoracoscope, there was no well-defined leak. We only detected a thinned diaphragm (Fig. 2b) and were unable to conclude that it was the responsible lesion. Therefore, we made a single laparoscopic port on the right side of abdomen near the navel and infused CO_2_ gas into abdominal cavity at 8 cmH_2_O. By inspecting the diaphragm with a thoracoscope under pneumoperitoneum, bubbles were seen gushing out from a small pore at the right central tendon of the diaphragm, which was considered the responsible lesion (Fig. 2c). The lesion was closed with 4-0 non-absorbable monofilament sutures (PROLENE, Ethicon, Somerville, NJ, USA) in a Z suture (Fig. 2d).

**Fig. 3:**
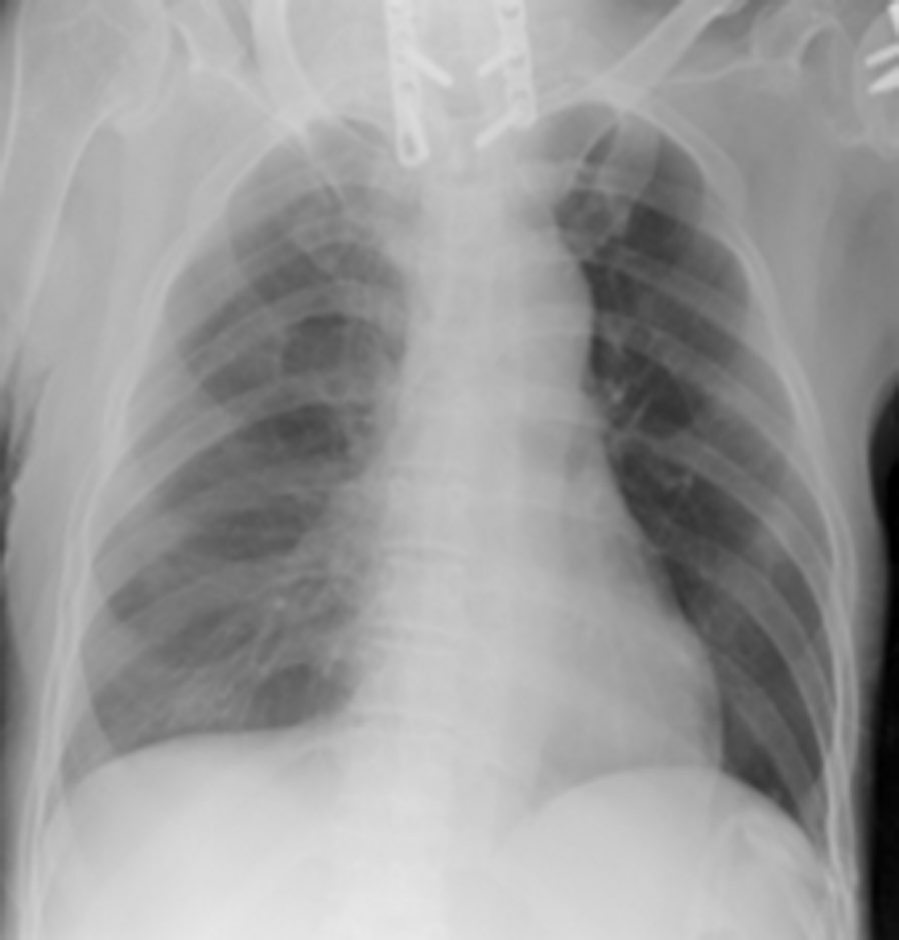
The hydrothorax was resolved after the operation despite the re-initiation of peritoneal dialysis

To avoid the induction of liver damage by suturing, the diaphragm was pulled enough and then sutured. After treatment, bubble leakage was no longer observed. The central tendon around the reinforcement was covered with a sheet of absorbable polyglycolic acid (PGA) felt (Neoveil, Gunze, Osaka, Japan) using fibrin glue (Beriplast P, CSL Behring, King of Prussia, PA, USA) (Fig. 2e). The patient resumed CAPD one week later with the same volume of dialysis as usually used; however, there was no recurrence of the hydrothorax for over one month (Fig. 3).

## Discussion

PPC occurs in 1.6% of patients who undergo CAPD, and the average interval between the start of dialysis and the occurrence of hydrothorax ranges from 1 day to 8 years [3]. Approximately half of these patients are forced to permanently switch from CAPD to hemodialysis [2]. Hydrothorax is usually right-sided and more prevalent in women than in men, involving cough, chest pain and dyspnea, although it can be asymptomatic as well [3].

The pathogenesis of hydrothorax in CAPD remains unclear. According to some studies, a PPC either through congenital or acquired diaphragmatic fenestrations and the leakage of fluids secondary to increased abdominal pressure is the most common explanation for the etiology of hydrothorax. The diagnosis is usually not difficult because of the close relationship between the increase in the pleural effusion and CAPD performance. Laboratory data can sometimes assist in its diagnosis as well. Chow et al. [4] reported recently that a pleural fluid-to-serum glucose concentration difference exceeding 50 mg/dl had 100% sensitivity and specificity for differentiating hydrothorax secondary to pleuroperitoneal communication from other causes. Similarly, Tang et al. [5] reported that a high concordance rate between the plural and peritoneal fluid protein content (uniformly < 4 g/l) could also be used as a reliable surrogate marker of pleuroperitoneal communication. Regarding the definitive diagnosis, dialysis solution containing dyes or contrast materials has been used for the detection of leakage, and recently, a radioactive isotope examination was performed for the decisive evaluation of this disease [3].

VATS is the standard procedure for treating this condition. However, Saito et al. reviewed 29 cases of PPC and found that there were 8 cases in which fistulas were unconfirmed during the operations, and in these cases, the recurrence rate was relatively high [2]. In such cases, although dialysis solution containing dye is used to detect fistulas, the responsible lesions cannot be confirmed during surgery. This is probably due to the pressure effect of the hydrothorax impeding the migration of the dye across the diaphragm. Therefore, it is necessary to increase the intra-abdominal pressure to a value above the intra-thoracic pressure. In our case, infusing CO_2_ gas and increasing the intra-abdominal pressure caused bubbles to gush out of a small pore, which confirmed that it was the lesion responsible for PPC. Although some previous studies have reported a similar approach using a CO2 pneumoperitoneum through a peritoneal dialysis (PD) catheter [6], we opted for making an additional port in the abdomen after taking such factors as safety and certainty into consideration. In fact, we experienced some cases in which we could not inject any substances into the abdomen due to obstructions associated with the PD catheter. The laparoscopic approach eliminates such concerns, although there remains insufficient evidence to definitively recommend utilizing a PD catheter as the main port for CO2 gas infusion. Finally, the laparoscopic approach provides surgeons with an excellent view of the diaphragm from the abdominal cavity.

Regarding the treatment of PPC, various approaches have been reported, such as mechanical rub pleurodesis and chemical pleurodesis using tetracycline, fibrin glue, steroid, *Nocardia rubra* cell wall skeleton, OK-432, and autologous blood [5]. Regardless of the treatment used, the desired pleural adhesion was not always firmly formed, resulting in the frequent recurrence of hydrothorax. We chose to perform direct sutures with VATS in the present case because we were able to access the lesion with good visualization under pneumoperitoneum. In this case, VATS combined with laparoscope was ideal approach.

## Conclusions

We herein report a case in which VATS combined with laparoscopic pneumoperitoneum was effective for the detection of the lesion responsible for PPC. For the accurate diagnosis and treatment, we recommend VATS combined with laparoscopy as the surgical method of first choice for PPC.

## Data Availability

Not applicable.

## Abbreviations

CT: Computed tomography
VATS: Video-assisted thoracic surgery
PPC: Pleuroperitoneal communication
CAPD: Continuous ambulatory peritoneal dialysis
PD: Peritoneal dialysis
